# Comparable peri‐transplant mortality and incidence of febrile neutropenia after high‐dose melphalan and autologous stem cell transplantation in patients with plasma cell dyscrasias treated as outpatient versus inpatient

**DOI:** 10.1111/bjh.70104

**Published:** 2025-09-02

**Authors:** Ana Maria Avila Rodriguez, Xiao Hu, Cindy Varga, Raymond Comenzo, Shayna Sarosiek, J. Mark Sloan, Vaishali Sanchorawala, Gheorghe Doros, Karen Quillen

**Affiliations:** ^1^ Department of Medicine Boston University Chobanian & Avedisian School of Medicine Boston Massachusetts USA; ^2^ Division of Hematology/Oncology University of Illinois at Chicago Chicago Illinois USA; ^3^ Division of Hematology‐Oncology Tufts Medical Center Boston Massachusetts USA; ^4^ MaineHealth Cancer Care Portland Maine USA; ^5^ Atrium Health Levine Cancer Institute Charlotte North Carolina USA; ^6^ Dana Farber Cancer Institute Bing Center for Waldenstrom's Macroglobulinemia Boston Massachusetts USA; ^7^ Department of Biostatistics Boston University School of Public Health Boston Massachusetts USA; ^8^ Department of Medicine Beth Israel Deaconess Medical Center Boston Massachusetts USA

**Keywords:** AL amyloidosis, multiple myeloma, stem cell transplantation

To the Editor,

High‐dose chemotherapy with autologous stem cell transplantation (ASCT) remains an important therapeutic option for selected eligible patients with multiple myeloma (MM) and immunoglobulin light‐chain amyloidosis (AL).^1^ With growth factor, antimicrobial prophylaxis and infrastructure support, outpatient ASCT has been possible since the 1990s. Outpatient ASCT in MM has comparable treatment‐related mortality, lower rates of febrile neutropenia and improved survival compared to inpatient ASCT,^2^, ^3^, ^4^ based on data from single‐centre observational studies which include strict selection criteria for outpatient or inpatient protocol within the institution. Outpatient ASCT in AL is less studied.

At one of our academic centres (‘A’), all patients undergoing ASCT for plasma cell dyscrasia are managed in the outpatient setting, unless admission is necessary for febrile neutropenia, gastrointestinal toxicity or other adverse events. Eligibility criteria for ASCT in general include Eastern Cooperative Oncology Group (ECOG) performance status ≤ 2, Left Ventricular Ejection Fraction (LVEF) ≥ 40%, diffusing capacity of the lungs for carbon monoxide (DLCO) > 50%, systolic blood pressure ≥ 90 and oxygen saturation on room air ≥95%. Patients with AL cardiac involvement are evaluated by a cardiologist with additional testing such as cardiopulmonary stress test as recommended. Patients with febrile neutropenia are hospitalized according to the American Society of Clinical Oncology (ASCO) guidelines with prompt initiation of intravenous antibiotics.^5^ At the other academic medical centre located in the same city (‘Centre B’), all ASCT patients with plasma cell dyscrasia are managed in the inpatient setting from conditioning regimen until engraftment.

Stem cell mobilization was performed in accordance with institutional protocols. In the inpatient cohort, patients had a temporary apheresis catheter for collection and a non‐tunnelled triple‐lumen central line placed just prior to the administration of conditioning chemotherapy. Patients in the outpatient cohort had a tunnelled apheresis catheter placed prior to mobilization; this catheter remained for the duration of the transplant. Supportive care peri‐transplant included growth factors and prophylactic antimicrobial therapy. Antifungal and antiviral prophylaxis was identical between cohorts. For antibiotic prophylaxis, oral ciprofloxacin was given in the inpatient cohort and levofloxacin in the outpatient cohort. The outpatient transplant infrastructure at Centre A includes daily visits to the treatment centre with a comprehensive physician evaluation on weekdays, and nursing visits on weekends (for laboratory assessment, as needed transfusions). Patients and caregivers were required to mask outside the home and treatment centre and live within 5 miles (cost of local short‐term housing covered by insurance or charitable support).

We retrospectively collected data on all consecutive patients who underwent ASCT for plasma cell dyscrasia at the two centres between January 2014 and December 2018, after institutional review board approval. The primary objective of our study was to compare peri‐transplant mortality (from conditioning to Day +100) and the incidence of febrile neutropenia between the two cohorts; secondary objectives included the incidence of bacteraemia and overall survival.

Data on 183 patients were analysed (103 outpatients and 80 inpatients). There were no significant pretransplant differences between the two cohorts except for diagnosis composition and extent of induction chemotherapy (see Table 1). Among patients transplanted for amyloidosis, 12/32 (37.5%) of the inpatient cohort and 35/63 (55.6%) of the outpatient cohort had not received any induction therapy prior to transplant. There was no significant difference in the proportion of amyloidosis patients with cardiac involvement between the two cohorts.

**TABLE 1:** Baseline patient characteristics.

	Inpatient (*n* = 80)	Outpatient (*n* = 103)	*p*
Age, mean (range)	59.6 (34–73)	58.1 (43–76)	0.245
Male (%)/female (%)	46 (58)/34 (43)	64 (62)/39 (38)	0.525
Diagnosis, *n* (%)			
Multiple myeloma (MM)	47 (58.8)	38 (36.9)	
Plasma cell leukaemia	1 (1.3)	2 (2.0)	
Light chain, heavy chain amyloidosis (AL‐AH) MM/AL, MM/AH	32 (40.0)	63 (61.2)	0.00697
eGFR < 45 mL/min	16 (20)	14 (13.6)	0.337
eGFR ≥ 45 mL/min	64 (80)	89 (86.4)	
Number of organs involved per patient with amyloidosis (median)	2	1	
Organ involvement in amyloidosis patients, *n* (%)			
Renal	20 (62.5)	54 (85.7)	0.021
Cardiac	15 (46.9)	24 (38.1)	0.574
Autonomic nervous system	4 (12.5)	5 (7.9)	0.728
Peripheral nervous system	6 (18.8)	5 (7.9)	0.223
Gastrointestinal	10 (31.2)	5 (7.9)	0.008
Liver	3 (9.4)	2 (3.2)	0.428
Skin soft tissue	1 (3.1)	14 (22.2)	0.034
Splenic	0 (0.0)	1 (1.6)	1.000
Hypofibrinogenaemia	0 (0.0)	1 (1.6)	1.000
Pulmonary	0 (0.0)	1 (1.6)	1.000
Vascular	1 (3.1)	0 (0.0)	0.729
Induction chemotherapy, *n* (%)			
None	12 (15)	35 (34)	0.0004222
<6 cycles	31 (39)	48 (46)	
≥6 cycles	30 (37)	18 (17)	
Prior high dose melphalan and SCT	7 (9)	2 (2)	

Abbreviation: SCT, stem cell transplantation.

Transplant metrics are shown in Table 2. More patients in the outpatient cohort (79% vs. 56%) received full high‐dose melphalan (200 mg/m^2^). There was no difference in the incidence of febrile neutropenia (47% outpatient vs. 54% inpatient) or peri‐transplant 100‐day mortality (1.9% outpatient vs. 2.5% inpatient) between cohorts. With a median follow‐up of 36.9 and 32.5 months, respectively, overall survival is 85% at 6 years for the inpatient cohort, compared to 76% for the outpatient cohort (HR = 0.6, 95% CI [0.3–1.5]).

**TABLE 2:** Stem cell transplantation metrics.

	Inpatient (*n* = 80)	Outpatient (*n* = 103)	*p*
Conditioning chemotherapy			
Melphalan dosing 200 mg/m^2^, *n* (%)	44 (56)	81 (79)	<0.001
Melphalan dosing 140 mg/m^2^	34 (43)	22 (21)	
CD34+ cell dose infused (10^6^/kg), mean (SD)	5.81 (2.62)	7.87 (3.25)	<0.001
Days to ANC engraftment, mean (SD)	12.05 (3.5)	10.26 (1.33)	<0.001
Febrile neutropenia (FN), *n* (%)	43 (54)	48 (47)	0.3374
Positive blood cultures, *n* (%)	7 (9)	24 (23)	0.00924
Associated with FN	5	20	
Not associated with FN	2	4	
eGFR < 45 mL/min	2	3	
eGFR ≥ 45 mL/min	5	21	
Inpatient days, median (range)	19 (12–40)	6.5 (1–37)	<0.001
Patients requiring ICU care, *n* (%)	4 (5)	20 (19)	<0.001
AL‐AH, MM/AL, MM/AH	1	15	
MM	3	5	
ICU days, median (range)	2.5 (2–4)	3 (1–20)	0.5006
Mechanical ventilation, *n*	0	3	
Vasopressors, *n*	2	6	
GI toxicity (any grade)	63 (78.8)	100 (97.1)	<0.001
Typhlitis (CT imaging), *n* (%)	1 (1.2)	3 (2.9)	0.800
Clostridium difficile infection, *n* (%)	2 (2.5)	3 (2.9)	1.000
Mortality Day + 100, *n* (%)	2 (2.5)	2 (1.9)	0.7978
Multiple myeloma	1 (2.1)	0	
Amyloidosis	1 (3.1)	2 (3.2)	

Species isolated in blood cultures	Inpatient (*n* = 7)	Outpatient (*n* = 24)^a^
Gram positives, *n* (%)	6 (86)	14 (58)
*Staphylococcus* species	3	6
*Streptococcus* species	3	3
Gram negatives, *n* (%)	1 (14)	16 (64)^b^
*Escherichia coli*	0	8

Viral organism isolated	Inpatient (*n* = 1)	Outpatient (*n* = 14)
Cytomegalovirus	1	0
Coronavirus (NL63, 229E, OC43)	0	3
Rhinovirus/enterovirus	0	4
Respiratory syncytial virus	0	2
Parainfluenza 2/3/4	0	4
Influenza	0	1

^a^ Five outpatients had blood cultures positive for more than one organism.

^b^ Gram‐negative organisms other than *E. coli* include Bacteroides species (*n* = 2), Leptotrichia wadei (*n* = 1), Capnocytophaga (*n* = 1), Pseudomonas aeruginosa (*n* = 1), Enterobacter cloacae (*n* = 1), Klebsiella pneumoniae (*n* = 1). One gram‐negative blood culture was not speciated.

While the majority of patients in the outpatient cohort required hospitalization during the peri‐transplant course (47% of patients for febrile neutropenia and 38% for other indications such as gastrointestinal toxicity or cardiac complications), the number of days in the hospital was much shorter among those who were hospitalized (6.5 days), compared to 19 days for the inpatient cohort. Gastrointestinal toxicity of any grade occurred in a higher proportion of patients in the outpatient cohort, although the majority of patients (83%) had grade 2 toxicity. There was no difference in typhlitis or C difficile infection. The incidence of bacteraemia was significantly higher in the outpatient cohort (23.3% vs. 9%), with a high burden of Gram‐negative bacteraemia. *Escherichia coli* was most frequently identified, and 87.5% of *E. coli* isolates were levofloxacin resistant. Organisms isolated in the inpatient cohort were predominantly Gram‐positive bacteria. More patients within the outpatient cohort required ICU admission: 22% of AL and 12.5% of myeloma patients at Centre A required ICU admission. Bacteraemia in the outpatient cohort was not associated with ICU admission: 5/24 (21%) of patients with bacteraemia and 15/79 (19%) of patients without bacteraemia required ICU admission at Centre A. The duration of ICU stay was similar between outpatient and inpatient cohorts (3.0 vs. 2.5 days respectively).

The current study is the first to compare peri‐transplant mortality, incidence of febrile neutropenia and bacteraemia in outpatient and inpatient protocols for ASCT in an autologous transplant population enriched for AL amyloidosis. In prior retrospective single‐institution studies comparing inpatient versus outpatient transplant in MM, patients with advanced disease and impaired pretransplant renal function were selected for inpatient ASCT and were more likely to receive melphalan dose reductions.^3^, ^6^ Our study is a two‐centre analysis of an unselected outpatient ASCT cohort, which matched the unselected inpatient cohort in age and renal function. Our study demonstrates that outpatient transplant for AL amyloidosis is feasible with low peri‐transplant mortality at a high‐volume centre. The peri‐transplant deaths at Centre A occurred in two patients with AL amyloidosis, on Day +43 (from refractory hypotension related to autonomic dysfunction) and Day +29 (acute hypoxic respiratory failure from diffuse alveolar haemorrhage), despite maximal intensive care support. At Centre B (inpatient cohort), one patient with myeloma died on Day +34 from pancolitis and one patient with amyloidosis died on Day +66 from renal and heart failure. The treatment‐related mortality (TRM) of the AL amyloidosis cohort at Centre A of 3.2% (2/63) is identical to the TRM reported in 2012–2020 in a recent multicentre retrospective review by Muchtar and colleagues.^7^, ^8^ While febrile neutropenia was the most common reason for hospitalization in the outpatient cohort, consistent with other reports,^9^, ^10^ the incidence of febrile neutropenia was not significantly different from that observed in the inpatient cohort.

The incidence of bacteraemia in the outpatient cohort, while higher than the inpatient cohort, is in line with the incidence of bacteraemia reported by Muchtar.^8^ Despite differences in bacteraemia, there were no differences in febrile neutropenia or peri‐transplant mortality between the cohorts in our study. Contrary to previous studies reporting Gram‐positive bacteraemia as the most common infection post‐ASCT in AL amyloidosis, the most common bloodstream infection (BSI) in our outpatient cohort was Gram‐negative bacteraemia from *E. coli* resistant to levofloxacin.^10^, ^11^ There is a retrospective single‐centre report of higher BSI in MM ASCT outpatients (24.7% vs. 12.9%), associated with multidrug‐resistant Gram‐negative organisms^6^; the authors hypothesize that outpatients may undergo more line manipulations. Differences in catheter care and duration of catheter placement between our cohorts may indeed be important factors. Monitoring institutional data for BSI should be an ongoing quality improvement metric at all transplant centres. There is no evidence to favour one specific fluoroquinolone (ciprofloxacin vs. levofloxacin) for peri‐transplant prophylaxis.^5^, ^12^ The role of more aggressive antimicrobial prophylaxis in outpatient ASCT such as intravenous antibiotics—adopted in some centres—is uncertain.^3^ The observation of higher rates of viral infection in our outpatient ASCT cohort in the pre‐COVID‐19 era suggests community contact exposure.

One explanation for the higher incidence of gastrointestinal toxicity and bacteraemia in the outpatient cohort is the increased use of unmodified high‐dose melphalan conditioning, which could lead to higher gut flora translocation into the bloodstream. Gastrointestinal involvement in AL patients did not appear to predict higher GI toxicity, as the outpatient AL amyloid cohort had lower rates of GI involvement. A higher rate of post‐transplant bacteraemia in ASCT for AL amyloidosis has been associated with impaired pretransplant renal function.^11^, ^13^ In our study, the proportion of patients with impaired renal function was similar between cohorts, although the outpatient AL cohort had a higher proportion of renal involvement (86% vs. 63%). There was no difference in bacteraemia stratified by renal function in either cohort (data not shown).

Outpatient transplants offer less deconditioning and lower costs. Only compliant patients (with caregivers) who can reliably report symptoms and present promptly for evaluation should be candidates.^3^ Outpatient ASCT is feasible for patients with AL amyloidosis and multiple myeloma at experienced transplant centres. Infrastructural support for outpatient ASCT provides the platform to implement outpatient immunotherapy protocols for select patients with haematological malignancies, such as CAR‐T‐cell therapy and bispecific antibody regimens. Directions for future study include patient‐reported outcomes and the impact of reserving ASCT to later lines of therapy in AL amyloidosis, after initial combination quadruplet chemoimmunotherapy.^14^, ^15^, ^16^

## AUTHOR CONTRIBUTIONS

A.R., X.H. and K.Q. designed the study, performed the research, analysed data, interpreted results and wrote the manuscript. G.D. analysed data and interpreted results. C.V., R.C., S.S., J.M.S. and V.S. edited the manuscript.

## FUNDING INFORMATION

This study was not funded.

## CONFLICT OF INTEREST STATEMENT

The authors do not have relevant conflicts of interest to declare.

## ETHICS APPROVAL STATEMENT

The study was approved by the Institutional Review Board at Boston University Medical Center and Tufts Medical Center.

## PATIENT CONSENT STATEMENT

Informed consent for prospective data collection was obtained in accordance with the Declaration of Helsinki.

## Data Availability

The data that support the findings of this study are available from the corresponding author upon reasonable request.
